# Antibody-drug conjugates targeting Trop-2: Clinical developments in early breast cancer therapy

**DOI:** 10.1016/j.breast.2022.10.015

**Published:** 2022-10-26

**Authors:** Jae Ho Jeong, Sung-Bae Kim

**Affiliations:** Department of Oncology, Asan Medical Center, University of Ulsan College of Medicine, Seoul, South Korea

**Keywords:** Early breast cancer, Antibody-drug conjugates, Trop-2, Sacituzumab govitecan, Neoadjuvant, Adjuvant, ADC, antibody-drug conjugate, Dato-DXd, datopotamab deruxtecan, DFS, disease-free survival, HER2, human epidermal growth factor receptor 2, HR, hormone receptor, iDFS, invasive disease-free survival, NACT, neoadjuvant chemotherapy, OS, overall survival, pCR, pathological complete response, PFS, progression-free survival, SG, sacituzumab govitecan, T-DM1, trastuzumab emtansine, TNBC, triple-negative breast cancer, TPC, treatment of physician's choice, Trop-2, Trophoblast cell surface antigen-2

## Abstract

Although breast cancer has a good prognosis compared with various cancers, metastatic breast cancer has an aggressive disease course and remains incurable. Therefore, treatment of early breast cancer to prevent recurrence and metastasis is crucial. Recently, the development of anti-cancer drugs, such as targeted agents and immuno-oncology, has been accelerating. Antibody-drug conjugates (ADCs) are also building a new paradigm. Particularly, ADCs targeting Trop-2 were approved for their efficacy in metastatic triple-negative breast cancer patients who received ≥2 prior systemic therapies and showed significant results in heavily pretreated hormone receptor-positive/HER2-negative breast cancer. In this brief review, we provide an overview of ongoing clinical trials of ADCs targeting Trop-2 in early breast cancer, specifically sacituzumab govitecan.

## Highlights

• Antibody-drug conjugates (ADCs) are building a new paradigm in cancer treatment.
• Sacituzumab govitecan (SG) and datopotamab deruxtecan (Dato-DXd) are ADCs targeting Trop-2.
• SG and Dato-DXd showed improved efficacy in metastatic breast cancer treatment.
• Several studies using SG are ongoing in the neoadjuvant/adjuvant setting for early breast cancer.

## 1 Introduction

Trophoblast cell surface antigen 2 (Trop-2), a transmembrane glycoprotein, is expressed in all subtypes of breast cancer, especially in more than 85% of triple-negative breast cancer (TNBC) [1], where its high expression is associated with a poor prognosis [2,3]. Antibody-drug conjugates (ADCs) targeting Trop-2 have shown clinical efficacy in various cancers [1,[4], [5], [6]], and many clinical trials of these drugs are ongoing. Among the Trop-2-targeting ADCs, sacituzumab govitecan (SG) and datopotamab deruxtecan (dato-DXd) are being actively explored for treating advanced/metastatic breast cancer [1,4,5]. As with the development of most drugs in oncology, those that show activity against advanced/metastatic cancer need to be explored to improve early breast cancer outcomes [[7], [8], [9], [10]]. SG improves overall survival in patients with heavily pretreated TNBC that is widely refractory to chemotherapy and has activity in high-risk or endocrine resistant hormone receptor (HR)-positive/human epidermal growth factor receptor 2 (HER2)-negative metastatic breast cancer [1,4,11]. SG was approved by the US Food and Drug Administration in 2020 and the European Medicines Agency in 2021 as an indication for the treatment of metastatic TNBC in patients who received at least two prior chemotherapy regimens for metastatic disease [12,13].

### 1.1 Mechanism of action

ADCs consist of three structures: a monoclonal antibody that binds to a specific tumor cell receptor, a cytotoxic agent (payload), and a chemical linker that connects the antibody and the payload. SG is composed of a monoclonal antibody targeting Trop-2 and SN-38 (an active metabolite of irinotecan), a topoisomerase I inhibitor, in a ratio of 1:7.6, and the antibody and payload are linked by the cleavable linker [14]. Preclinical investigation of SG showed that increased Trop-2 expression was significantly associated with SG's therapeutic activity [15]. Since Trop-2 is also expressed in normal cells, there were concerns about toxicity in the drug development stage. A dose escalation study in cynomolgus monkeys (which express Trop-2 in similar tissues as humans) was performed [16]. Even with the highest dose of SG, most of the monkeys exhibited side effects due to SN-38, and as a result of histological examination, only minimal toxicity was seen in Trop-2-expressing tissues, with recovery by the end of the study.

After SG binds to Trop-2, it is internalized into the cell, and then SN-38 is released. Additionally, because it is a cleavable linker, SN-38 is released in the tumor microenvironment [14]. Due to this mechanism of cleavable linkers, SG has a bystander effect [14,[17], [18], [19]].

SN-38, a topoisomerase I inhibitor, is administered as irinotecan, a prodrug form, and is mainly metabolized by the liver. However, only a small portion of irinotecan is converted to SN-38, so its bioavailability is limited [20]. Preclinical research was conducted to compare the concentrations of SN-38 via SG in tumor tissue and normal tissues using a xenograft model, compared with irinotecan [21]. SG delivered 20- to 136-fold more SN-38 to the tumor than irinotecan, and it reduced intestinal uptake, showing a favorable profile in terms of toxicity. This study demonstrated why SG using the same SN-38 as payload was more effective, in terms of drug delivery, than irinotecan.

A recent study using a multiscale quantitative pharmacokinetic approach demonstrated SG to have efficient tumor penetration and rapid payload release [22]. Unlike other ADCs that use ultratoxic drugs, SG uses SN-38, a more moderately toxic agent, so tissue penetration can be improved due to high ADC dosing (10 mg/kg). Additionally, a second dose after 1 week may improve tissue penetration from residual antibodies in circulation.

The advantage of ADCs is that their therapeutic indices (ratio of toxicity to tumor vs. normal cells) are higher than those of cytotoxic chemotherapeutics, and SG has less toxicity than SN-38 in terms of pharmacokinetics and half-life [14,23]. The half-life of SG is approximately 11–14 h, and it releases nearly 90% of SN-38 in 3 days [24]. SN-38 of SG is mostly bound to IgG, so the ratio of SN-38G, a glucuronidated form, is low [25]. Therefore, the enterohepatic recirculation of SN-38G is reduced, and the risk of diarrhea is lowered.

In this context, ADCs targeting Trop-2 are also being studied in patients with high-risk early breast cancer. Currently, there are four studies on SG for early breast cancer, but none on dato-DXd. Herein, we briefly describe major ongoing clinical trials of SG for early breast cancer, which are summarized in Table 1.

**Table 1:** Ongoing clinical trials of sacituzumab govitecan for treating early breast cancer.

Reference	Study design	No. of patients	Target disease	Treatment setting	Treatment	Primary outcome	Recruitment status	Estimated Primary Completion Date	ClinicalTrials.gov identifier
SASCIA trial	Phase IIIRCT	1200 (1:1 allocation)	HER2-negativeearly breast cancer	Adjuvant	1. Experimental arm: SG (8 cycles) 2. TPC arm: capecitabinecarboplatin or cisplatin observation	iDFS	Recruiting (October 2020-)	December 2026	NCT04595565
NeoSTAR trial	Phase IIsingle arm	51	Early TNBC	Neoadjuvant	SG 10 mg/kg D1 & D8, every 3 weeks (4 cycles)	pCR	Completed enrollment	October 2024	NCT04230109
ASPRIA trial	Phase IIsingle arm	40	Early TNBC	Adjuvant	SG + atezolizumab (6 cycles)	Rate of undetectable circulating tumor cfDNA- 6 cycles	Recruiting (July 2020-)	December 2023	NCT04434040
COGNITION-GUIDE trial	Phase IIseven-arm umbrella	240	Early breast cancer	Adjuvant	Arm 1: AtezolizumabArm 2: InavolisibArm 3: IpatasertibArm 4: OlaparibArm 5: SGArm 6: Trastuzumab/pertuzumabArm 7: observation	iDFS	Not yet recruiting	April 2029	NCT05332561

RCT, randomized controlled trial; SG, sacituzumab govitecan; TPC, treatment of physician's choice; iDFS, invasive disease-free survival; TNBC, triple-negative breast cancer; pCR, pathologic complete response.

## 2 SASCIA trial (NCT04595565)

**Fig. 1:**
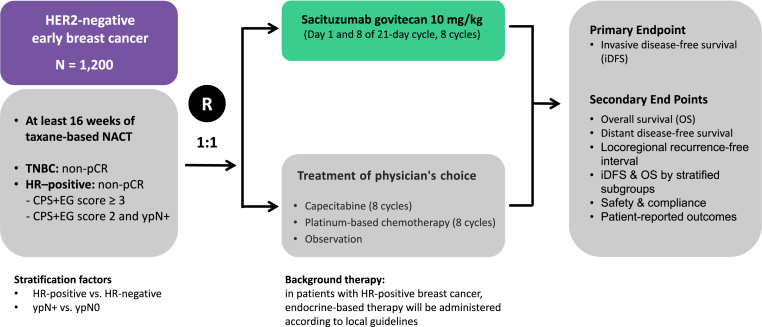
Scheme of the clinical design of the SASCIA trial [26].

SASCIA is an ongoing phase III randomized trial that is enrolling patients with HER2-negative breast cancer with residual disease after neoadjuvant chemotherapy (Fig. 1). Patients must have received neoadjuvant taxane-based chemotherapy for at least 16 weeks (anthracyclines are permitted). The participants are randomly assigned in a 1:1 ratio to receive SG at a dose of 10 mg/kg (days 1 and 8, every 3 weeks for 8 cycles) or treatment of their physician's choice (TPC): capecitabine (1000 mg/m^2^ twice daily on days 1–14, every 3 weeks for 8 cycles), carboplatin (AUC 5, every 3 weeks for 8 cycles; or AUC 1.5, weekly for 24 weeks), cisplatin (75 mg/m^2^ every 3 weeks for 8 cycles; or 25 mg/m^2^ weekly for 24 weeks), or observation. For patients with HR-positive breast cancer, endocrine-based therapy will be administered according to local guidelines. The primary endpoint is invasive disease-free survival (iDFS).

The results of an interim safety analysis were presented at the European Society for Medical Oncology Breast Cancer 2022 meeting [26]. In total, 88 patients (SG, n = 45; TPC, n = 43 (capecitabine, n = 32; observation, n = 11)) were included in the analysis. For all patients assigned to the TPC group, capecitabine was the only drug used, which was probably based on the results of the EA1131 study [27]. Grade 3/4 adverse events were observed in 66.7% of the SG group and 28.1% of the capecitabine group. Dose reduction occurred in 31.1% (14/45) of the SG arm and 40.6% (13/32) of the capecitabine group. Therapy discontinuation occurred in 13.6% of the SG and 9.4% of the capecitabine groups. This trial is expected to be completed by 2026.

NACT; neoadjuvant chemotherapy; pCR, pathological complete response; TNBC, triple-negative breast cancer; HR, hormone receptor; HER2, human epidermal growth factor receptor 2; CPS-EG, clinical and pathologic stage and estrogen receptor status and histologic grade; ypN+, pathologically lymph node positive; iDFS, invasive disease-free survival; OS, overall survival.

## 3 NeoSTAR trial (NCT04230109)

**Fig. 2:**
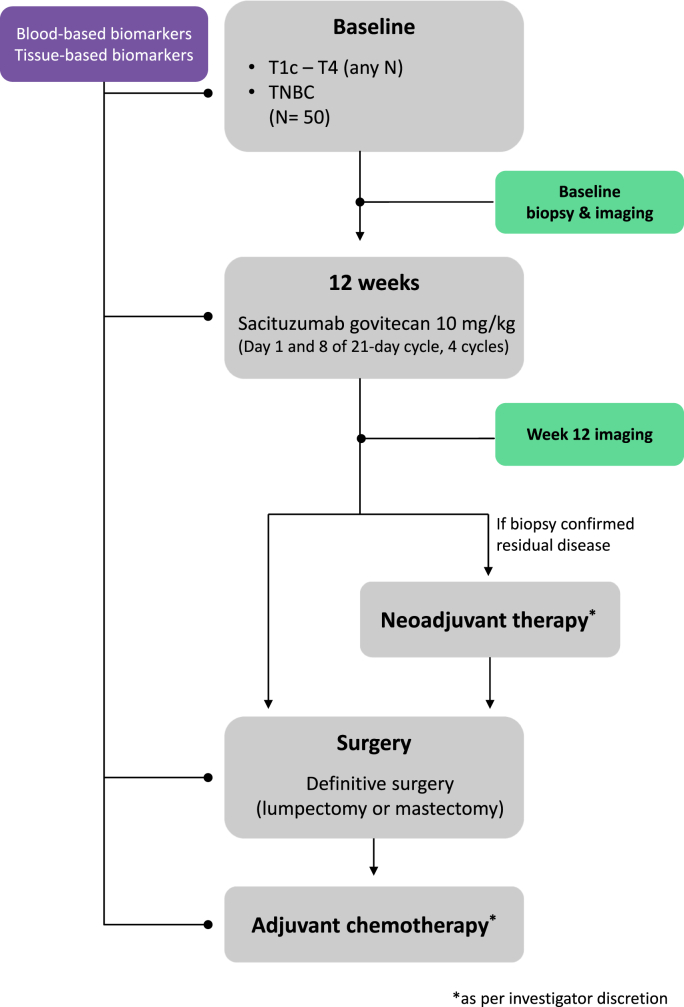
Scheme of the clinical design of the NeoSTAR trial [28]. TNBC, triple-negative breast cancer.

NeoSTAR was a phase II trial evaluating neoadjuvant SG for patients with localized TNBC (tumor size ≥1 cm or any size if node-positive) (Fig. 2). The primary endpoint was the pathological complete response (pCR, ypT0/isN0) rate with SG. Secondary objectives included radiological response rate assessments, safety and tolerability evaluations (CTCAE ver. 5.0), and event-free survival. Four cycles of neoadjuvant SG (10 mg/kg, days 1 and 8, every 3 weeks) were administered, after which a biopsy was performed, and the primary endpoint (pCR) was confirmed. In the event of non-pCR, additional neoadjuvant treatment was allowed at the discretion of the treating physician.

The NeoSTAR results were presented as a poster discussion session at the 2022 American Society of Clinical Oncology annual meeting [28]. Of 50 patients enrolled in the study, 98% (49/50) completed 4 cycles of SG. More than half of the 50 patients (n = 26) proceeded directly to surgery after SG. The pCR rate with SG alone was 30% (n = 15; 95% CI: 18%, 45%). Of the 24 patients who received additional neoadjuvant therapy after SG, 25% (n = 6) achieved pCR. The pCR rate in eight patients with BRCA mutation was 75%. As patients with BRCA mutations have difficulty with repairing double strand breaks and the payload SN-38 for the SG is actually worked by increasing single strand DNA breaks, it would make sense that those with the BRCA mutation might be particularly benefiting from SG. The most common adverse events with SG were nausea, fatigue, alopecia, neutropenia, anemia, and rash. None of the patients discontinued SG treatment due to disease progression or adverse events. Further research on the optimal duration of SG, as well as on neoadjuvant combination strategies, including immunotherapy, is needed.

## 4 ASPRIA trial (NCT04434040)

ASPRIA is an ongoing single-arm phase II trial evaluating SG and atezolizumab in combination as adjuvant treatment for patients with TNBC who have residual invasive disease after neoadjuvant therapy. The rationale behind combining an ADC with checkpoint blockade is as follows: T cells enter the tumor microenvironment in response to ADC binding, and their antitumor activity is markedly increased by cytotoxic T-lymphocyte–associated antigen 4 (CTLA4) and programmed death-ligand 1 (PD-L1) inhibition. Both innate and adaptive immune mechanisms exhibit antitumor activity against cells expressing the ADC target antigen as well as adjacent cancer cells in the tumor microenvironment [29]. Patients will receive 6 cycles of adjuvant therapy composed of a combination of SG (days 1 and 8, every 3 weeks) and atezolizumab (day 1, every 3 weeks). The primary endpoint is the rate of undetectable circulating tumor cell-free DNA after 6 cycles (at 18 weeks). In total, 40 patients will be enrolled. The trial is expected to be completed in 2023.

## 5 COGNITION-GUIDE trial (NCT05332561)

COGNITION-GUIDE is a 7-arm umbrella phase II trial evaluating genomics-guided post-neoadjuvant therapy in high-risk breast cancer patients with residual cancer. In this biomarker-driven trial, eligibility for the respective study arms is determined by the molecular tumor board. Inclusion into the respective arms is determined by the following biomarkers:

• Arm 1 (atezolizumab, immune evasion): PD-L1 positivity measured using immunohistochemistry (≥1% on immune cells within the tumor), microsatellite instability-high status (measured by polymerase chain reaction), tumor mutational burden-high (≥10 mut/MB), cluster of differentiation 274 (CD274) amplification
• Arm 2 (inavolisib, phosphatidylinositol-3-kinase (PI3K)): known/reported oncogenic mutation in PIK3Ca
• Arm 3 (ipatasertib, protein kinase B (AKT)): aberrations predicting increased PI3K-AKT pathway activity, except PI3K mutations, HR-positive histology
• Arm 4 (olaparib, poly ADP ribose polymerase [PARP]): inactivating somatic or germline BRCA1/2 mutation
• Arm 5 (SG, Trop-2): Trop-2 overexpression (measured using immunohistochemistry and except known/reported homozygous polymorphism in UGT1A1*28)
• Arm 6 (trastuzumab/pertuzumab, HER2): HER2 exon-20 insertion, activating HER2 mutation

The primary endpoint is iDFS at 4 years. In total, 240 patients will be enrolled. Presently, patient recruitment has not yet begun.

## 6 Compliance: how can we mitigate SG adverse events and avoid early treatment dropout?

Notably, in the interim safety analysis of the SASCIA trial, neutropenia (any grade, 82.2% vs. 37.5%), diarrhea (46.7% vs. 28.1%), and alopecia (68.9% vs. 15.6%) were more predominant in the SG group than in the capecitabine group. The treatment dropout rate is usually higher in association with early breast cancer than with metastatic breast cancer. For example, the rates of T-DM1 discontinuation due to adverse events were 7.0% in the TH3RESA study (metastatic breast cancer) and 18.0% in the KATHERINE study (early breast cancer). Likewise, the rates of SG discontinuation due to adverse events were 5.0% in the ASCENT study (metastatic breast cancer) and 11.0% in the SASCIA study (early breast cancer). As the genetic polymorphism UGT1*28/28 is associated with a higher risk of grade ≥3 neutropenia but not diarrhea, systematic pre-therapeutic UGT1A1 genotyping or the primary prophylactic use of granulocyte colony-stimulating factor might be considered in some cases. Better management of SG side effects is crucial, given that the agent will not be used as a standalone treatment.

## 7 Future directions

More effective strategies are needed for patients with post-neoadjuvant residual disease, especially those with TNBC and high-risk luminal breast cancer. The Trop-2-targeting agents need to be further explored in combination with existing agents (concurrently or sequentially) in high-risk patients. The benefits of SG in advanced TNBC and high-risk luminal breast cancer are expected to have better efficacy in early breast cancer settings.

Better selection strategies are also needed to find patients who will be at a higher risk of developing grade ≥3 adverse events, especially neutropenia, to optimize the risk/benefit ratio of ADCs in TNBC and luminal breast cancer treatment. Investigations of SG in combination with immune checkpoint inhibitors (NCT04538742, NCT04468061), DNA damage and repair inhibitors (NCT04644068, NCT03992131, NCT04039230), and endocrine therapies (NCT04556773, NCT04553770) are ongoing in metastatic setting, which ultimately will be introduced into earlier setting. A better understanding of new combination strategies based on solid scientific rationale and demonstration of their strong efficacy will clear the way toward a cure for breast cancer.

## Funding

None.

## Declaration of competing interest

JH Jeong has received honoraria from AstraZeneca, Boryung Pharmaceutical, Eisai, Lilly, Kyowa Kirin, Novartis, Pfizer, and Roche. He has been a consultant on advisory boards for Boryung Pharmaceutical, Eisai, Lilly, Novartis, and Pfizer.

SB Kim received research funding from 10.13039/100004336Novartis, Sanofi-Aventis, and DongKook Pharm Co.

He has been a consultant on advisory boards for Novartis, AstraZeneca, Lilly, Dae Hwa Pharmaceutical Co. Ltd, ISU Abxis, OBI Pharma, Beigene, and Daiichi-Sankyo. He owns stocks of Genopeaks and NeogeneTC.
